# Drug-induced mitochondrial toxicity: Risks of developing glucose handling impairments

**DOI:** 10.3389/fendo.2023.1123928

**Published:** 2023-02-13

**Authors:** Auxiliare Kuretu, Charles Arineitwe, Mamosheledi Mothibe, Phikelelani Ngubane, Andile Khathi, Ntethelelo Sibiya

**Affiliations:** ^1^ Pharmacology Division, Faculty of Pharmacy, Rhodes University, Makhanda, South Africa; ^2^ School of Laboratory Medicine and Medical Sciences, University of KwaZulu-Natal, Durban, South Africa

**Keywords:** diabetes mellitus, insulin resistance, mitochondria, toxicity, glucose

## Abstract

Mitochondrial impairment has been associated with the development of insulin resistance, the hallmark of type 2 diabetes mellitus (T2DM). However, the relationship between mitochondrial impairment and insulin resistance is not fully elucidated due to insufficient evidence to support the hypothesis. Insulin resistance and insulin deficiency are both characterised by excessive production of reactive oxygen species and mitochondrial coupling. Compelling evidence states that improving the function of the mitochondria may provide a positive therapeutic tool for improving insulin sensitivity. There has been a rapid increase in reports of the toxic effects of drugs and pollutants on the mitochondria in recent decades, interestingly correlating with an increase in insulin resistance prevalence. A variety of drug classes have been reported to potentially induce toxicity in the mitochondria leading to skeletal muscle, liver, central nervous system, and kidney injury. With the increase in diabetes prevalence and mitochondrial toxicity, it is therefore imperative to understand how mitochondrial toxicological agents can potentially compromise insulin sensitivity. This review article aims to explore and summarise the correlation between potential mitochondrial dysfunction caused by selected pharmacological agents and its effect on insulin signalling and glucose handling. Additionally, this review highlights the necessity for further studies aimed to understand drug-induced mitochondrial toxicity and the development of insulin resistance.

## 1 Introduction

Diabetes mellitus (DM) is a metabolic disorder characterized by hyperglycaemia due to defects in insulin secretion, insulin action, or both (1, 2). Researchers have drawn much attention to DM, lately due to the rapid increase in mortality and related complications. According to the International Diabetes Federation (IDF), DM has become one of the major threats to human health. At present, there are 537 million adults (20-79 years) living with DM according to the latest data from the (IDF) (2, 3). This figure is expected to grow to 643 million by 2030 and 783 million by 2045, respectively. Approximately, 6.7 million deaths were related to DM in 2021. Most cases of diabetes fall into two broad etiopathogenetic categories, these include type 1 and type 2 diabetes (1–3). Type 1 diabetes mellitus (T1DM) is insulin-dependent and develops because of pancreatic beta-cell destruction which leads to insufficient insulin secretion. Type 2 (T2DM) which is also known as Non-Insulin Dependent Diabetes Mellitus (NIDDM), or adult-onset diabetes is prevalent (1–3). Often, the development of T2DM is attributed to the inability of insulin-sensitive cells to respond to insulin (1–3). According to clinical and non-clinical studies, T2DM accounts for approximately 90% of diabetes cases. The risk factors of T2DM include obesity, age, epigenomics, and lifestyle patterns (1–3).

Studies have established that insulin signalling underpins normal mitochondrial function in the metabolism of skeletal, liver, cardiac muscles, and pancreatic ß cells (4). The regulation of metabolic fuel homeostasis is associated with insulin signalling in different organs and tissues (1, 4). Insulin acts through a receptor found on the membrane of target cells. Insulin has pleiotropic effects in most cells for instance the brain (4). However, the major effects are found in the liver where the conversion of glucose into glycogen is promoted, and glucose output is decreased (4). The other important targets include the skeletal muscle and adipose tissue where insulin stimulates glucose uptake through translocation of GLUT4 (4, 5). GLUT4 is one of 13 human glucose transporter isoforms (GLUTs) with 12 membrane-spanning domains. GLUT4 is highly expressed in skeletal muscle and adipose tissue (4, 5). The translocation is achieved through a series activation of protein downstream of the insulin receptor. Several studies have reported on the dysfunction of insulin signalling pathway at various levels thus leading to poor glucose uptake, termed insulin resistance. Insulin resistance has been ascribed to several factors, including increased intramuscular fatty acids, hence insulin resistance models have been developed using palmitic acid exposure. Additionally, insulin resistance has been linked to inflammation and several studies have used tumour necrosis factor alpha (TNF-α)- to develop insulin resistance *in vitro.* Emerging evidence suggests that mitochondrial impairments may also potentiate insulin resistance development (5–7). Therefore, this review seeks to provide insights and potential links between mitochondrial toxicity and insulin resistance development. Also, we will spend some time discussing the potential development of insulin resistance through mitotoxic pharmacological agents. For a greater understanding of this narrated review presented, we will first outline and describe the insulin signalling pathway, looking at potential sites where disturbances could occur.

## 2 Insulin signalling pathway

Insulin regulates many pathways at molecular levels such as lipid synthesis and storage (liver and adipose tissue), stimulation of protein synthesis (in skeletal muscle and liver), inhibition of ketogenesis and gluconeogenesis (liver), and stimulation of glycolysis and glucose storage (skeletal muscle and liver) (7–9). Insulin increases energy utilization or storage by regulating the transport of glucose into the cell, this process is facilitated by GLUT 4, particularly in the skeletal muscle and adipose tissue. Glucose uptake mainly increases when the concentration of GLUT 4 proteins at the plasma membrane is enriched (8, 9). Insulin in the signal transduction pathway is mediated through the insulin receptor (IR) found on the cell membrane (9, 10). IR-mediated signal transduction can be divided into two pathways which are the IRS-mediated signal transduction pathway and the non-IRS-mediated signal transduction pathway.

The insulin receptor is a heterotetrametric transmembrane glycoprotein that is composed of α2β2 and is a tyrosine kinase receptor (9–11). The α and β subunits are linked by disulphide bonds. The α subunits are located extracellularly and this is where insulin binds. The β subunits span the cell membrane and consist of tyrosine-protein kinase subunits which are flanked by two regulatory regions (12–15). These two regions consist of phosphotyrosine-binding (PTB) sites where initiation of the insulin-signalling cascade occurs. When insulin binds to its cognate receptor, the closure of the α subunits causes β subunits to move closer to each other. This alters the conformation of the receptor leading to autophosphorylation of a specific tyrosine residue located on the β subunit. Subsequently, the kinase activity of the phosphorylated tyrosine ß subunit is increased (14–16). Insulin-receptor substrates (IRS1 and IRS2) are mostly expressed in humans although a series of IRS1-4 proteins are recruited and phosphorylated. IRS1 and IRS2 are expressed in the heart, skeletal muscle, brain, liver, adipocytes, and mammary gland where they play a significant physiological role. The phosphorylated tyrosine IRS-1 protein recruits and binds to an SRC homology 2 (SH2) domain which contains signalling molecules. These molecules activate two signal transduction pathways in the cell, PI3K/AKT and MAPK pathway (14–17).

### 2.1 Phospho-inositol 3 kinase/protein kinase B pathway

**Figure 1:**
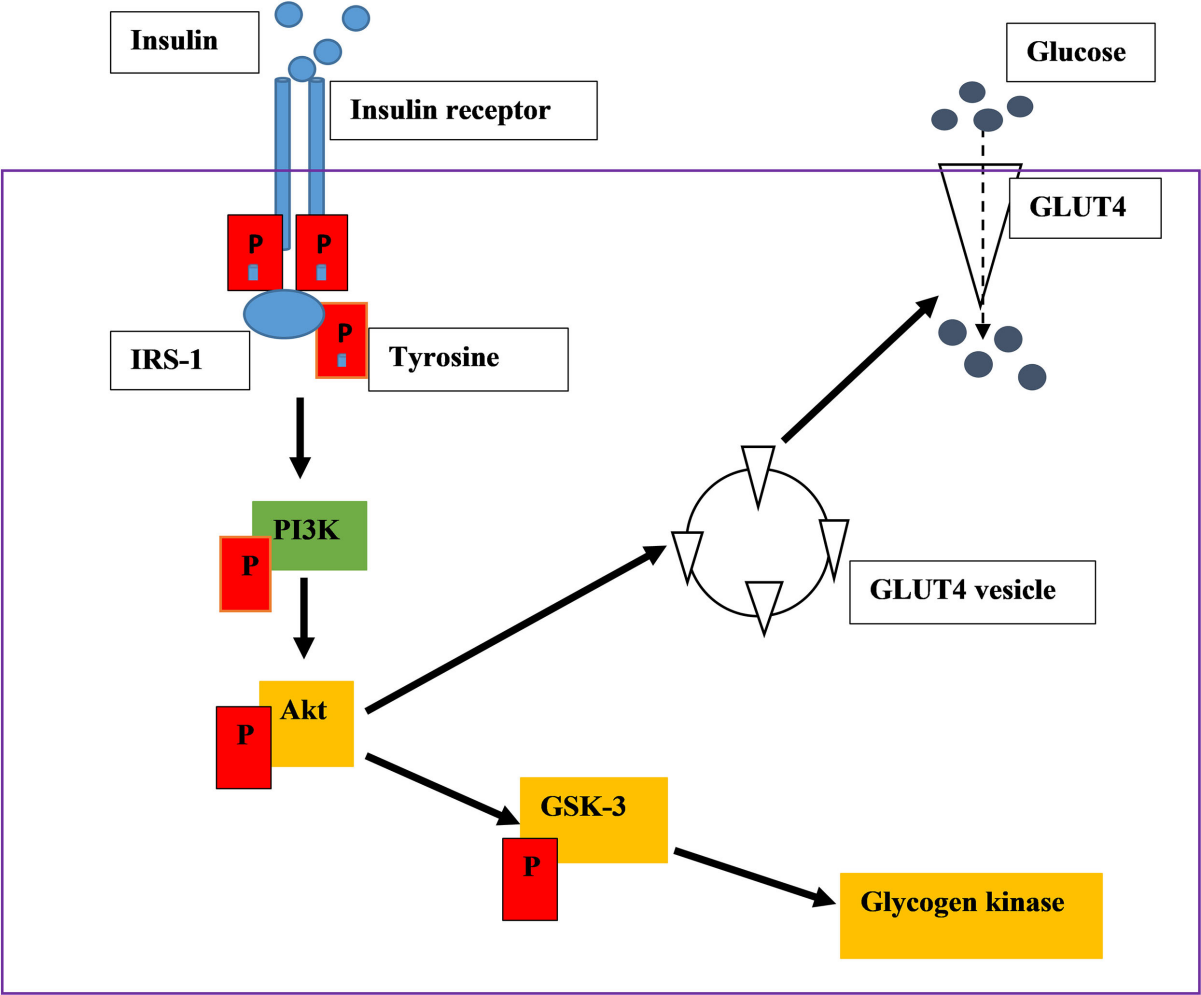
An illustration of insulin signalling pathway. Insulin binds on the insulin receptor extracellularly. The binding activates tyrosine kinase, leads into the phosphorylation of downstream kinases, ultimately stimulating GLUT 4 translocation which absorb glucose.

PI3K is a heterodimeric protein, which consists of two subunits. The p85 regulatory subunit consists of the SH2 domain and is involved in IRS-PI3K interaction. They also have the p110 subunits, which consist of the catalytic subunit of the enzyme (14–17). The phosphorylated IRS-1 protein acts as an attachment point to the P85 subunit of PI3K. P85 recruits its catalytic subunit P110 to activate P13K (14–17). PI3K activates the phosphorylation of phosphatidylinositol 4,5-bisphosphate [PI(4,5)P2] and 3,4,5-phosphatidylinositol-3,4,5-triphosphate (PIP3) is produced (14, 15, 17).This results in the activation of serine/threonine-protein kinase B (PKB/Akt) leading to the activation of many substrates. PKB is not membrane-bound therefore it can diffuse around the cell (15, 17). PKB stimulates the movement of glucose membrane transporters (GLUT4) to the cell membrane and activates enzymes that catalyse the conversion of glucose into glycogen (15, 17). For instance, when AS160 (Akt substrate of 160 kDa) is mono-phosphorylated, GLUT 4 is translocated to the cell membrane for glucose uptake (**Figure 1**). Glycogen synthase kinase 3(GSK3) phosphorylation inhibits its activity leading to the action of glycogen synthase. Therefore, the cellular uptake of glucose is promoted, and the synthesis of glycogen increases thus lowering the blood glucose concentration (14–17).

### 2.2 Ras-mitogen-activated protein kinase pathway/growth signalling pathway

The MAPK families include c-Jun N-terminal kinase (JNK), p38 and extracellular-signal-regulated kinase (ERK) in mammals (14, 17, 18). IRS 1 and IRS2 activate MAPK through differentiation by interacting with the PI3K-AKT pathway, binding to growth factor receptor-bound protein 2(Grb2), and regulating gene transcription and cell proliferation (14, 15, 17). IRS1 and IRS2 mediate lipid and glycogen synthesis respectively in the liver. Insulin IRS2-mediated signalling pathways are impaired more by insulin resistance compared to IRS1-mediated signalling pathways (14–16).

### 2.3 Pathophysiology of insulin resistance

The metabolic action of insulin is to maintain glucose homeostasis by suppressing glucose production in the liver and by promoting glucose uptake in the skeletal muscle and adipose tissue (2–4). A decreased sensitivity to these metabolic actions is known as insulin resistance (2–4). Insulin resistance is a biochemical abnormality that precedes T2DM and other associated diseases (1–3). Insulin resistance is characterised by the failure of insulin to stimulate glucose uptake by peripheral tissues or suppress hepatic glucose production (2, 3). This results in hyperinsulinemia, dyslipidaemia, and hyperglycaemia. A group of systemic disorders is induced when glucose and lipid metabolism is dysregulated. Therefore, underpinning molecular, and cellular mechanisms of insulin resistance is important in understanding the pathogenesis of diseases associated with IR such as DM (2–5).

Various factors such as genetics, stress, lack of exercise, and aging contribute to insulin resistance (2, 5). Glucose and lipid metabolism disorders lead to defects in insulin signalling which is linked to numerous pathological conditions. The circulating free fatty acids (FFAs) are elevated by excessive energy intake, stress, and lipodystrophy (14, 18). An elevation in plasma FFAs concentration leads to the accumulation of diacylglycerol (DG), FFA’s, and triglycerides in non-adipose tissue, including skeletal muscle, liver, heart, and β-cells (14, 18, 19). In previous research studies conducted, results indicated that high-fat feeding and lipid infusions in human subjects and rodents reduced insulin-stimulated glucose disposal (18, 19). These results suggest that impairment of the insulin signalling pathway due to defects in lipid metabolism is the major cause of insulin resistance. Impaired insulin signalling does not only affect insulin-stimulated glucose metabolism in skeletal muscle but also impairs insulin action in diverse tissues such as the liver, heart, vasculature, and adipose tissue (14, 18, 19). Also, numerous studies have demonstrated that insulin resistance can develop through exposure to high palmitic acid concentration (20).

## 3 Mitochondria and metabolism of substrates

Mitochondria are double-membraned organelles that surround a space known as a matrix. The matrix consists of various enzymes (oxidative phosphorylation enzymes) and their genome, mitochondrial DNA (mtDNA) (4–7). The inner membrane harbours various enzymes and acts as a barrier that is poorly permeable to various molecules (6, 7). Therefore, this membrane consists of transporters that allow endogenous compounds such as fatty acids, ADP, glutathione, pyruvic acid, and possibly xenobiotics (6, 8). Mitochondria consists of approximately 1500 predominantly nuclear-encoded proteins in addition to 13 proteins encoded by their DNA (mtDNA) (18, 19). The mtDNA in humans consists of 37 genes that encode 22 transfer RNAs and 2 ribosomal RNAs (12S and 16S rRNA), these all play a crucial role in maintaining mitochondrial function (18, 19).

The systemic glucose and lipid metabolism converge into the mitochondria where most of the cellular energy (ATP) is generated by coupling the tricarboxylic acid cycle (TCA) cycle with oxidative phosphorylation (OXPHOS) (7–9). Acetyl-CoA generated from fatty acid β-oxidation (lipids) and glycolysis (glucose) enters the TCA cycle in the mitochondrial matrix whereby the substrates are oxidized with the formation of CO_2_, FADH_2_, and NADH (8–21). The electrons from NADH and FADH2 are taken up by complexes I and II, respectively. They are then passed to complex III and IV through ubiquinone (Q) and cytochrome c (C). Molecular oxygen accepts electrons and is converted to water at complex IV. During the redox process, complexes I, II, and IV pump protons from the matrix into the mitochondrial intermembrane space (IMS) (19, 21). This creates an electrochemical gradient (membrane potential) and generates ATP through complex IV. ATP and other mitochondrial factors accomplish the coupling of glucose metabolism to insulin secretion in the pancreatic β cell (8, 9, 19).

The mitochondrial function is regulated at different levels, these include mitochondrial biogenesis, post-translational modification of the mitochondrial protein, mitochondrial dynamics, and super complex formation (7–9). Previous research studies observed that all these processes are dysregulated in individuals who have type 2 DM. Mitochondria impairment is associated with aging and pathological states such as diabetes, cancer, sepsis, and obesity (8, 9). Conversely, some of the pharmacological agents used in the management of these conditions can lead to mitochondrial function impairment (8–10). Mitochondrial damage is associated with a decrease in oxidative capacity and an increase in oxidative stress.

## 4 Mitochondrial dysfunction

Mitochondria dysfunction was first described in the 1960s and research has advanced to understand the role played by the mitochondria in health and disease (8–10). Some studies have discovered that mitochondria impairment plays a significant role in the pathogenesis of a wide range of disorders such as cardiomyopathy, fibromyalgia, and diabetes mellitus (8–10). Fuel metabolism is profoundly altered by DM, and both insulin resistance and insulin deficiency are characterised by excessive reactive oxygen species (ROS) production and inefficient mitochondria coupling (9, 10). The mitochondria have diverse roles in various cellular processes hence there are different definitions of mitochondria dysfunction (7, 10, 11). Some scientists refer to mitochondria dysfunction as diminished mitochondrial activity and oxidative phosphorylation (21–23). Others focus on different aspects such as the generation of oxygen species (9, 10).

### 4.1 Mechanisms of mitochondrial dysfunction

Mitochondrial impairment is mainly caused by reactive oxygen species (ROS) which are generated by the mitochondria (12–17). The current evidence states that complexes I (NADH: ubiquinone oxidoreductase) and III (ubiquinol: cytochrome c reductase) generate most of the ROS. This is likely due to the release of electrons by FADH and NADH into the ETC (19–22). Mitochondria consume approximately 85% of the oxygen utilized by the cell during ATP production (11). During normal oxidative phosphorylation (OXPHOS), about 0.4 – 4.0% of all oxygen consumed is converted to superoxide radical ( ${\text{O}}_{2}^{-}$ ) in the mitochondria. The superoxide is converted to hydrogen peroxide (H_2_O_2_) by the detoxification enzymes copper/zinc superoxide dismutase (Cu/Zn SOD) or manganese superoxide dismutase (MnSOD) (7, 21). The H_2_O_2_ is converted to water in the peroxiredoxin III (PRX III) or glutathione peroxidase (GPX) (7, 21). However, the ROS species accumulate in the mitochondria when the enzymes fail to catalyse these processes. This results in oxidative damage in the mitochondria (12). *In vitro* studies have observed that superoxide’s damage the iron-sulphur cluster that is found in the active site of aconitase, an enzyme in the TCA cycle (12, 13). This exposes the iron thus reacting with H_2_O_2_ to produce hydroxyl radicals through a Fenton reaction (12, 13). Nitric oxide (NO) is also produced within the mitochondria by mitochondrial NO synthase (mtNOS) and diffuses into the mitochondria from the cytosol (12, 14). The NO reacts with ${\text{O}}_{2}^{-}$ to produce a radical known as peroxynitrite (ONOO^−^) (12, 14). The cellular components that are most susceptible to free radicals in the mitochondria include the mtDNA, lipids, proteins, and OXPHOS enzymes (8–14). When mitochondrial proteins are damaged, their affinity to substrates or coenzymes is reduced thus decreasing their function (14). Mitochondrial damage can be precipitated by several chemical constituents including pharmacological therapies and environmental toxicants. In this era, where the use of pharmacological agents has been heightened, it’s of paramount importance to investigate its effect on mitochondrial function. Mitochondria impairments have been linked to the development of several disorders such as diabetes, cardiovascular diseases, and Alzheimer’s disease. Currently, the gold standard to measure mitochondrial content is transmission electron microscopy (TEM) (15, 16). Since, we have provided the foundation, the remaining part of this review focus on the potential contribution of drug-induced mitochondrial toxicity and the development of insulin resistance.

### 4.2 Mitochondrial dysfunction and insulin resistance

Individuals with insulin resistance can develop T2DM when pancreatic ß-cells are impaired, and they fail to secrete sufficient insulin to maintain euglycemia (7, 8). Oxidation of glucose by the mitochondria produces ATP thus increasing the ATP/ADP ratio. The ATP/ADP ratio is primarily regulated by mitochondrial function. An increase in ATP/ADP ratio is associated with the inhibition of the potassium channel (K_ATP_), this results in plasma membrane depolarization, the opening of voltage-gated calcium channel, an influx of calcium, and insulin secretion (7, 8). Therefore, the mitochondrial function can be correlated with the function of ß cells because of the importance of the ATP/ADP (7–10). Additionally, when mitochondrial genes are removed from ß cells secretion of insulin is impaired. The functionality of the ß cells is restored when the cells are replenished with mitochondria (12, 13). Results in previous research studies supported the notion that mitochondrial function plays a significant role in maintaining ß cell function (13–15). Thus, mitochondrial dysfunction is associated with T2DM pathogenesis by affecting the secretion of insulin and its action (15, 16).

Functional mitochondrial impairment is associated with the accumulation of lipids such as diacylglycerol and ceramide due to decreased fatty acid utilization (20–23). Dysregulation of lipid metabolism results in the accumulation of lipotoxic bodies that induce oxidative stress to the skeletal muscle and the liver (21, 23). Therefore, reduced mitochondrial oxidative capacity leads to an increase in intracellular lipid accumulation (23, 24). The skeletal muscles of type 2 diabetic patients have been reported to contain fewer mitochondria compared to those of age-matched insulin-sensitive individuals. Nuclear magnetic resonance spectroscopy studies showed a significant reduction of mitochondrial oxidative and phosphorylation activity in skeletal muscle and liver tissues of insulin-resistant individuals (6, 8). Additionally, mitochondrial impairment was observed in individuals with age-associated insulin resistance (5, 6). This was indicated by a reduction in insulin-stimulated skeletal muscle glucose uptake and/or metabolism and approximately a 40% reduction in the mitochondrial OXPHOS activity (5, 6). The findings from previous research studies strongly suggest that there is a correlation between insulin signalling and the normal functioning of the mitochondria in metabolism (5, 13, 14).

**Figure 2:**
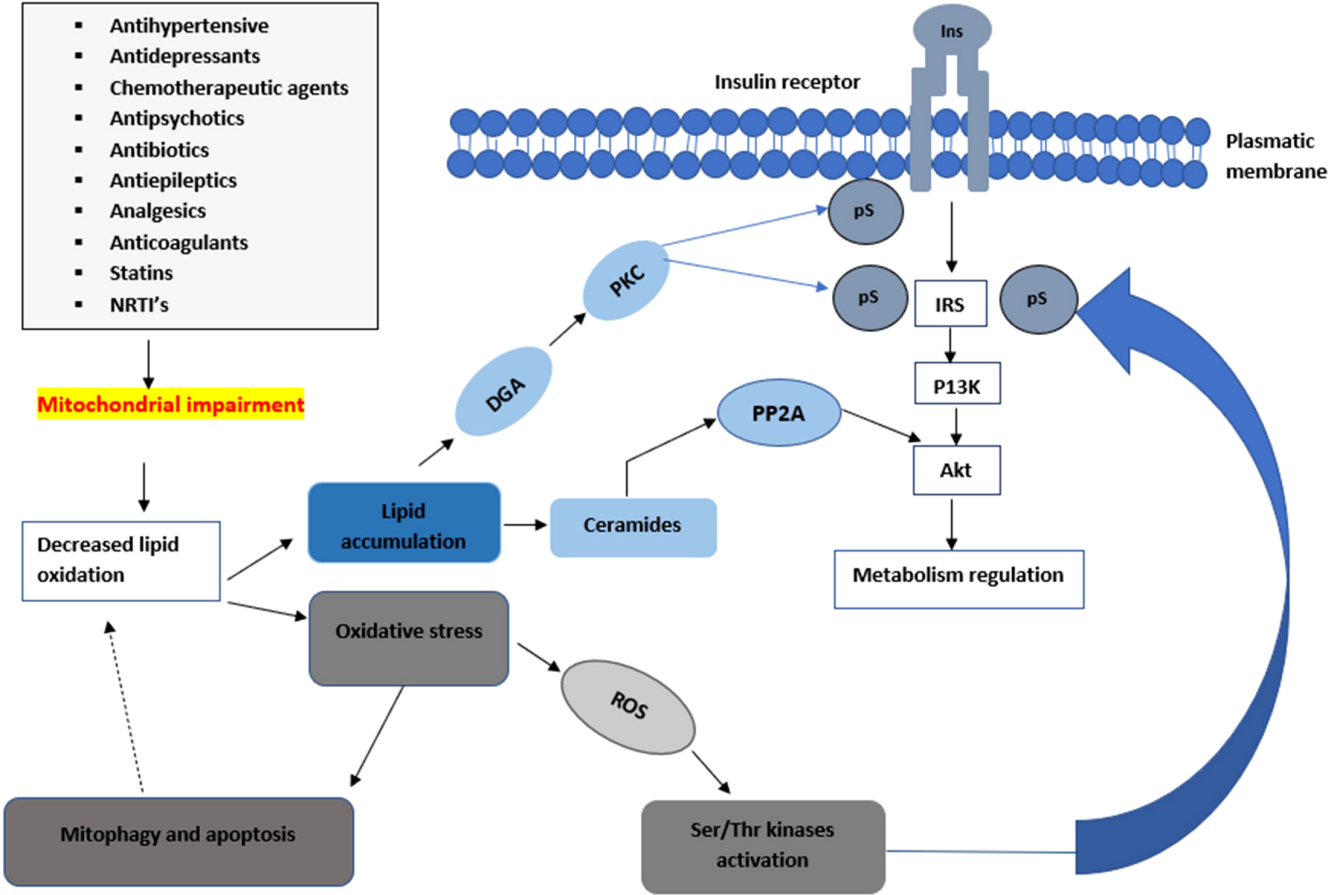
An illustration of how mitochondrial impairments can lead to a decrease in insulin sensitivity. Mitochondrial toxicity can lead to a decrease in the metabolism of lipids leading to the accumulation of ceramides and diacylglycerol which lead into the impairment of the insulin signalling pathway. The elevation of these metabolites can lead to a disruption of the insulin signalling pathway through the activation of stress-sensitive kinases such as PKC, ikappaB kinase IKK, and JNK. These stress-sensitive kinases can phosphorylate and inactivate IRS1 and IRS2.

A noteworthy observation demonstrated that insulin-resistant geriatric patients have significantly high triglyceride concentrations in both the skeletal muscle and liver cells (25). This implies that insulin resistance might arise due to defects in fatty acid oxidation in the mitochondria. These defects can increase toxic intracellular fatty acid metabolites (ceramide, fatty-acyl-CoA, and diacylglycerol) (**Figure 2**). The elevation of these metabolites can lead to a disruption of the insulin signalling pathway through the activation of stress-sensitive kinases such as protein kinase C (PKC), ikappaB kinase (IKK), and (c-Jun N-terminal kinases) JNK (11, 13). These stress-sensitive kinases can phosphorylate and inactivate IRS1 and IRS2. However, this concept is controversial, for instance, endurance-trained athletes have elevated levels of intramuscular triglycerides, yet they are highly insulin sensitive (22, 23). Mitochondrial capacity is substantially increased by aerobic exercise however insulin sensitivity is not improved (22, 24). Previous *in-vivo* studies conducted suggest that excess lipid concentration increases mitochondrial fatty acid oxidative capacity in the muscle of rodents (24). However, in some studies, a high-fat diet led to insulin resistance despite an increase of mitochondria in the skeletal muscles (26). The lack of congruence in these studies could be attributed to the complexities and differences in the experimental conditions used during the studies.

### 4.3 Drug-induced mitochondrial dysfunction and glucose handling impairments

**Figure 3:**
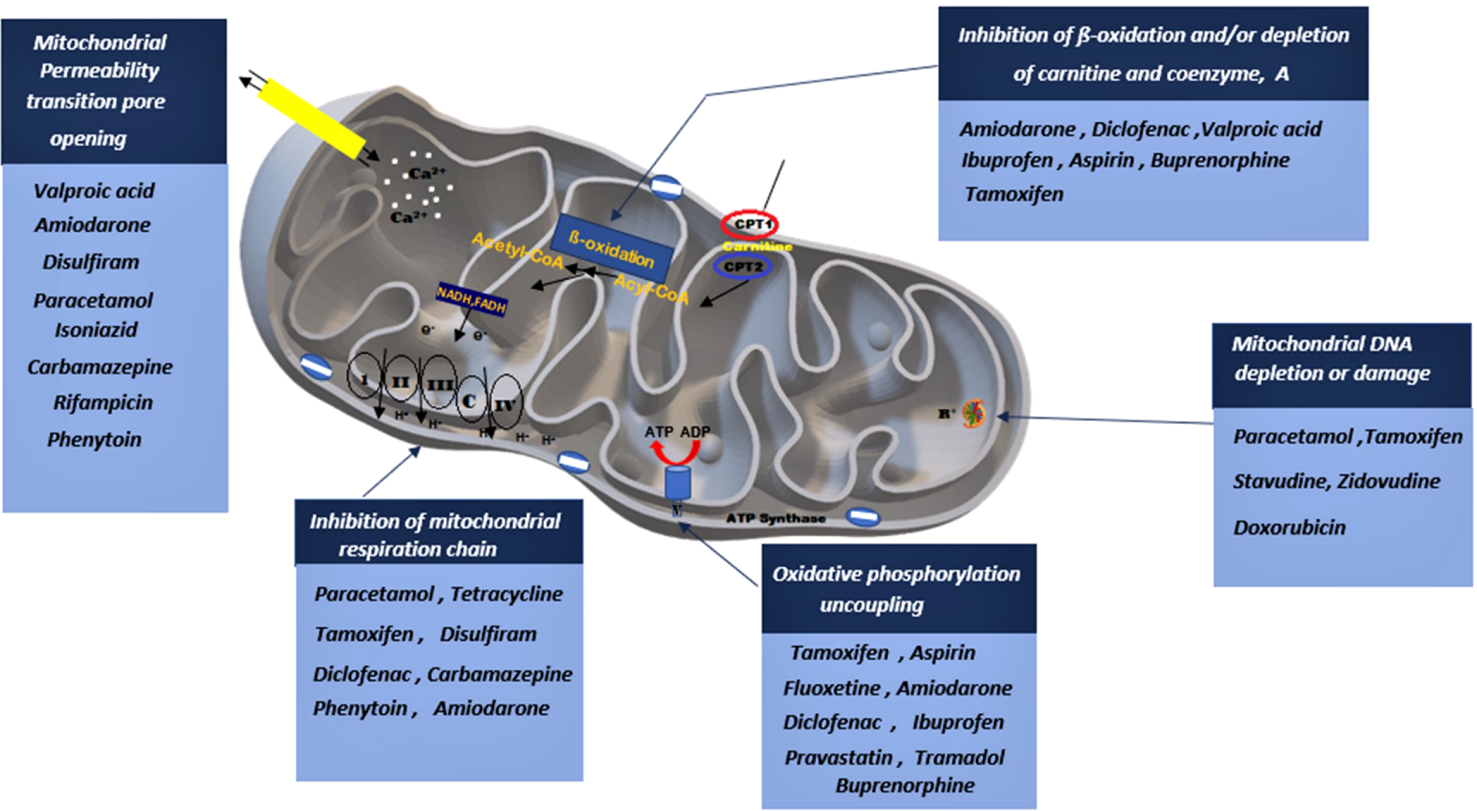
A depiction on how various drug classes can potentially lead to mitochondrial impairment through interfering with various mitochondrial sites. Drugs can stimulate the opening of the mitochondrial permeability pore, inhibit the mitochondrial respiratory chain, stimulate phosphorylation uncoupling, block β-oxidation and induced mitochondrial DNA damage.

Previous research studies reported well-established evidence of mitochondrial toxicity due to commonly used pharmacological agents (**Figure 3**). Knowledge of these adverse drug-mitochondrial interactions is important in assisting clinicians to design safer therapeutic regimens. Mitotoxic agents can directly exert their effect on the mitochondria by inhibiting mitochondrial DNA [mtDNA] transcription, electron transport chain [ETC], and ATP synthesis (18–22). Whilst other agents cause indirect toxicity by increasing the generation of ROS resulting in membrane depolarization, decreased antioxidant production, and mtDNA mutation (8, 9, 11). The mitochondrial permeability transition pores (MPTP) can open in some pathophysiological circumstances leading to the loss of structural and functional integrity of the membrane. There are several drugs and toxic compounds which induce the opening of the MPTP (18, 19). This significantly alters the mitochondrial structure, and function and exerts adverse effects on cell life. The exact pathway in which the cell is destroyed (by apoptosis and necrosis) depends on the number of mitochondria that are harbouring opened MPTP (19, 21). ATP synthesis is profoundly altered due to the loss of integrity of the inner mitochondrial membrane (9, 11). Drug therapy has many beneficial effects hence it is used to treat different ailments and improve symptom management (17–20). However, some pharmacological therapies have emerged to cause long-term side effects. Clinical manifestation of the adverse effects depends on the specific drug and the effect it exerts on the mitochondria. Since mitochondrial toxicity is induced through multiple pathways, different methods are required to evaluate mitotoxicity (18–20). Other than drug-induced mitochondrial toxicity, environmental toxins also have a significant impact on the mitochondria. Environmental toxins include compounds such as food additives, pesticides, plasticizers, flame retardants, and waste products. Humans are exposed mainly through ingestion, dermal uptake, and inhalation (25, 26). Mitochondrial toxicity is associated with several drug classes, however, patient-patient variability in mitochondrial function significantly impacts the response (21–23). For instance, patients who are diagnosed with mitochondrial disorders, particularly those carrying C1494T and A1555G mutations in human mitochondrial 12S rRNA, are acutely sensitive to aminoglycoside antibiotics, which can result in deafness. Drug classes such as statins, anti-diabetics, anti-epileptics, NSAIDs, anti-depressants, and certain antibiotics have been identified to induce mitochondrial toxicity (13, 18, 27), **Table 1** summarizes how different drug classes affect mitochondrial function and insulin sensitivity. Most of the studies conducted were performed in isolated cells and mitochondrial extract (24, 67). In this section, we will highlight mitotoxic effect of selected drugs and potential insulin resistance development.

**Table 1:** Summarizes the impact of known mitotoxic agents on insulin sensitivity .

DRUG CLASS	DRUGS	MITOCHONDRIAL EFFECTS	EFFECT ON INSULIN SENSITIVITY	REFERENCE
NRTI’s	Zidovudine	inhibits the polymerase that replicates mtDNA, thus preventing mitochondrial replication.	Decreases insulin sensitivity	(21, 28)
	Stavudine	MtDNA depletion or damage. Mitochondrial toxicity which induces lipodystrophy	Decreases insulin sensitivity	(29, 30)
Statins	Simvastatin	reduce coenzyme Q10 (CoQ10) levels	Decreases insulin sensitivity	(31)
	Pravastatin	Reduces coupling between electron transport chain and oxidative phosphorylation	Impairs insulin signalling and decreases insulin sensitivity	(32, 33)
Antipsychotics	Chlorpromazin	Inhibits ATPase activit	Exacerbates hepatic insulin sensitivity	(34)
	Haloperidol	Increases formation of mitochondrial ROS and mitochondrial mass	Increase insulin secretion	(35–37)
Antidepressants	Amitriptyline	Induces ROS formation	Decreases insulin sensitivity	(38)
	Sertraline	Perturbs mitochondrial membrane and causes swelling	Increases pancreatic insulin secretion	(36)
Chemotherapy agents	Doxorubicin	Increases ROS, mtDNA adduct and iron overload	Impairs systemic insulin sensitivity	(39)
	Cisplatin	Impairs ETC and increase ROS formation.	Impairs insulin secretion.	(38, 40)
	Tamoxifen	Inhibits mitochondrial respiration and elevated mitochondrial lipid peroxidation.	Decreases hepatic insulin sensitivity	(41, 42)
Antibiotic	Clarithromycin	inhibits the mitochondrial protein synthesis	Improves insulin sensitivity	(28, 31)
	Doxycycline	inhibits the mitochondrial protein synthesis	Improve insulin sensitivity	(43, 44)
Antihypertensives	carvedilol	Inhibits mitochondrial oxygen consumption Mild uncoupling of mitochondrial oxidative phosphorylation	Increases insulin sensitivity	(45–47)
	Captopril	Decreases mitochondrial ATP synthase activity	Improves hepatic insulin sensitivity	(48, 49)
Anti-epileptics	Valproic acid	Inhibits the TCA cycle and mitochondrial fatty acid oxidation	Enhances insulin resistance	(50, 51)
	Phenytoin	Inhibits ETC	Inhibits insulin release	(52, 53)
	Carbamazepine	Inhibits mitochondrial respiration.	Improves insulin sensitivity	(52, 54)
Analgesics	Paracetamo	Induce oxidative stress leading to mtDNA depletion or damage	Improves insulin secretion	(55–58)
	Tramadol	Directly inhibits mitochondrial fatty acid oxidation. OXPHOS uncoupling	Enhances hepatic insulin sensitivity	(59–61)
	Diclofenac	Increases mitochondrial oxidative stress	Improves insulin sensitivity	(60–62)
Anticoagulants	Aspirin	Uncouples mitochondrial oxidation.	Improves insulin sensitivity	(63–66)
	Clopidogrel	Decreases mitochondrial membrane potential Increase ROS production	Improves insulin sensitivity	(61)

#### 4.3.1 Nucleoside reverse transcriptase inhibitors antiviral therapy

Nucleoside reverse transcriptase inhibitors (NRTIs) antiviral therapy is known for successfully controlling the viral load. NRTIs have proven to have played a significant role in the reduction of mortality and morbidity of numerous viral infections such as human immunodeficiency virus (HIV) (18, 19, 68). However, there is a risk of NRTI-induced inhibition of human DNA polymerases (27, 67). This is due to structural conservation between human and viral polymerases and the disruption of dNTP pools. A well-supported hypothesis ‘DNA pol-γ hypothesis of mitochondrial dysfunction is believed to be caused by polymerase inhibition (19, 20). NRTI treatment inhibits DNA pol-γ in mitochondria and this leads to mtDNA depletion. DNA pol-γ is the only known DNA polymerase that is found in the mitochondria and is responsible for the replication and repair of mtDNA (67, 68). It was discovered that early HIV therapy led to substantial levels of toxicity such that approximately 25% of the patients defaulted due to either toxicity or non-compliance (29, 69). Other HIV drugs such as zalcitabine have been completely withdrawn from the market because it was associated with mitochondrial toxicity (69). The mitochondrial toxicity observed is accompanied by lipodystrophy and cardiovascular risks. Fleischman et al. (70) used detailed physiological techniques to provide novel information on the effects of stavudine, administration on insulin sensitivity and mitochondrial function. This study demonstrated significant changes in insulin sensitivity by the hyperinsulinemic-euglycemic clamp in response to stavudine (70). Furthermore, in a descriptive and prospective study Judith et al. (30) demonstrated that the long-term use of ART contributes to insulin resistance. NRTIs have been shown to induce insulin resistance by inducing endoplasmic reticulum (ER) stress and reducing glucose transporter 4 (GLUT4) translocation to the plasma membrane in the skeletal muscle and adipose tissue (71). The NRTIs also inhibit mitochondrial DNA-polymerase, respiratory chain function, and ATP production, ultimately leading to adipocyte death. A study has attributed a decrease in insulin sensitivity to increase in lactate plasma levels upon NRTIs administration (71). Lactate administration has been directly shown to diminish insulin sensitivity in *in vitro and in vivo* studies (32). Since lactate accumulation is associated with mitochondrial dysfunction, one may suggest that NRTIs induced insulin resistance is linked with mitochondrial toxicity. As mentioned above, NRTI depletes mtDNA, thus having a negative impact on mitochondrial function such as OXPHOS enzymes. Nevertheless, studies are still required to demonstrate the direct mitochondrial toxicity on insulin resistance development risks associated with NRTIs.

#### 4.3.2 Cholesterol-lowering agents

Statin therapy (HMG-CoA reductase inhibitors) lowers cholesterol concentration and has become the most prescribed class of drugs worldwide for preventing cardiovascular diseases and treating hypercholesterolemia (33, 72). Statins competitively inhibit 3-hydroxy-3-methylglutaryl CoA (HMG-CoA) reductase, an enzyme that catalyses the conversion of HMG-CoA to mevalonic acid, the precursor of cholesterol (33, 72). This results in the reduction of endogenous cholesterol synthesis. Generally, statins are well tolerated, however, new emerging evidence suggests that long-term statin therapy is associated with adverse effects that may occur, for example, new onset of diabetes mellitus which is associated with impaired ß cell function, insulin resistance, or a combination of both processes (72, 73). Previous research studies discovered that patients on statin therapy showed oxidative stress, reduction in GLUT-4 and coenzyme Q10 (CoQ10) expression, reduced respiratory enzyme activity, and calcium leakage which led to mitochondrial-induced myopathies (32, 33, 71–73). Statin therapy is associated with skeletal muscle symptoms such as fatigue and wasting in hypercholesterolemia mice *in vivo*. These observations were attributed to impairments of the phospho-inositol/phosphate kinase B pathway. As mentioned above, this pathway is crucial for insulin sensitivity in target cells. Statin-induced muscle dysfunction and insulin resistance development were proven to be related to mitochondrial dysfunction through increased apoptotic markers and oxidative stress (32, 72). Also, chronic treatment with pravastatin led to ß cell dysfunction associated with increased beta-cell oxidative stress, reduced insulin exocytosis, and death (72). Although mitochondrial dysfunction is acknowledged as one way in which statins can induce insulin resistance, however, other mechanisms have been proposed. Statins can also negatively impact isoprenoid synthesis thus decreasing insulin sensitivity in adipocytes (74). Isoprenoids have demonstrated to play a role in the insulin signalling pathway. Additionally other studies have demonstrated that statin exposure inhibits adipocyte maturation and GLUT 4 expression (75). These observations clearly demonstrate that insulin resistance development because of statin therapy involves other pathways apart from the mitochondrial dysfunction.

#### 4.3.4 Chemotherapeutic agents

Chemotherapeutic agents such as anthracyclines and kinase inhibitors are known for their adverse effects. They are highly potent to ensure that they combat the highly resistant cancer cells (26, 27, 72). However, most chemotherapeutic agents induce the generation of excessive ROS which leads to oxidative stress and mitochondrial damage (6, 8). For instance, doxorubicin is an anthracycline antitumor antibiotic that is used to treat a wide range of solid tumours and haematological malignancies (76, 77). However, doxorubicin induces mitochondrial dysfunction, this might be due to increased nitric oxide production, accumulation of iron in mitochondria, and ROS production due to futile redox cycling (42). Recent studies have revealed the development of insulin resistance in patients receiving doxorubicin. Upon further investigations, it was found that this chemotherapeutic agent impairs the AMPK pathway, ultimately leading to a decreased GLUT 4 expression in the skeletal muscle (76). In this study, the AMPK expression was not affected, but a lack of phosphorylation was observed, suggesting impairments in the activation of the enzyme. Additionally, a decreased expression of IRS-1 and GSK-b was observed *in vitro*, and it was accompanied by an increase in ROS and complex I and II dysfunction, which could be implicating mitochondrial toxicity (42). In clinical settings. it has been observed that serious hyperglycemia associated with doxorubicin increase patient’s stay in the hospital and delays wound healing. Yang et al. (77) analyzed risk factors associated with doxorubicin-induced hyperglycemia in patients, and in this study, it was found, the risk of hyperglycemia was increased further by using corticosteroids. Tamoxifen is a highly lipophilic chemotherapeutic agent which exerts its anticancer effects by inducing apoptosis and oxidative stress *via* mechanisms that involve the mitochondria (41, 42). High concentrations of tamoxifen between 20 to 100µmol/L inhibited mitochondrial permeability transition pore opening in isolated rat liver mitochondria (41). Additionally, these high concentrations increased mitochondrial respiration by disrupting the mitochondrial membrane integrity and decreased oxidative phosphorylation and mitochondrial transmembrane potential (Δ*ψ*) (41). Tamoxifen has been reported to increase the incidence of diabetes mellitus (41). Hepatic steatosis in rodents and a decrease in pancreatic ß-cell survival and proliferation have been attributed to this potential adverse effect (42). Klöting et al. (41) *in-vivo* study demonstrated that upon administration tamoxifen caused approximately 75% loss of body fat followed by a phase of weight gain within the first weeks. The weight regain led to the accumulation of ectopic fat and the development of fatty liver. Importantly the redistribution of fat was associated with insulin sensitivity impairment both at hepatic and systemic levels (41). Cisplatin is a widely prescribed chemotherapeutic agent for the treatment of paediatric and adult tumours (38, 40). However, cisplatin has been reported to accumulate in the mitochondria forming adducts with mtDNA and proteins (38, 40). Cisplatin-induced hyperglycemia was reported by Komdeur et al. (40) in a 43-year-old patient who was receiving 100mg/m^3^ intravenously once every 4 weeks. The patient did not have a history of pre-existing glucose intolerance and was admitted in a lethargic state 6 days after his cisplatin third cycle. The patient was in a severe state of hyperglycaemic hyperosmolar (40). Although chemotherapy is associated with glucose handling impairments, the direct involvement of mitochondria toxicity remains elusive. This can prove useful in identifying the target site to reserve glucose metabolism dysregulation induced by chemotherapy.

#### 4.3.5 Antipsychotics

Mitochondrial adverse effects are also observed with antipsychotic drugs. These agents have been used since the 1950s for psychotic disorders such as mood disorders and schizophrenia with the development of chlorpromazine as the first anti-psychotic (5, 6, 78). Adverse effects originating from the mitochondria became apparent as metabolic disturbances caused by swelling and mitochondrial membrane depolarization were observed (5, 21). Sodium valproate an epileptic drug with well-documented mitochondrial toxicity shows respiratory dysfunction in HEPG2 cells *in-vitro* (21). Antipsychotics have also shown to negatively affect mitochondrial function. An *in vitro* study conducted by Cikánková et al. (78) has demonstrated that typical and atypical antipsychotics decrease mitochondrial metabolic rates in pig brains as evidenced by reduced oxygen consumption. In this study, chlorpromazine, haloperidol, zotepine, quetiapine, risperidone, and clozapine were associated with a decrease in the mitochondrial complex I, III, and IV activity with less inhibitory activity of citrate synthase (78). However, antipsychotics have been recognized to elicit weight gain and metabolic disturbances, many studies have demonstrated the obesogenic nature of various antipsychotics as they stimulate appetite and weight gain, ultimately metabolic syndrome. Studies have shown that antipsychotics inhibit receptors involved in suppressing hunger such as H1 and 5HT receptors (79). As a result, chronic overfeeding behaviour because of antipsychotics leads to dysregulated lipid profile, ultimately insulin resistance. To date, the involvement of mitochondrial dysfunction remains unclear in the development of insulin resistance associated with antipsychotic therapy. However, from a biochemical and pharmacological perspective, it makes sense to associate these disturbances with a decreased activity of mitochondrial function. A randomized clinical trial conducted by Nicol and colleagues demonstrated a decrease in insulin sensitivity after 12 weeks of treatment with olanzapine, aripiprazole, and risperidone associated with abdominal and subcutaneous fat deposition (80). Subsequent studies have also revealed that antipsychotics exert insulin resistance independent of weight gain associated with metabolic syndrome. A study by Teff et al. (81) demonstrated that atypical antipsychotics blunt insulin sensitivity in the absence of weight gain in healthy individuals. This was the first study to suggest a direct effect of antipsychotics on insulin-sensitive cells. The mechanisms underlying the development of insulin resistance are not clear and may manifest at various levels of insulin signalling. However, the primary defect appears to be a markedly attenuated ability to phosphorylate the post-receptor insulin receptor substrate (82). Hence, studies on the effect of exposure to antipsychotics on insulin-sensitive cells in a glucose-handling context are critical. Such studies would shed light on the direct impact of antipsychotics on insulin sensitivity with hopes of elucidating associated mechanisms, and a direct implication of mitochondrial toxicity. Understanding the role of mitochondrial toxicities associated with antipsychotics could perhaps be key in managing glucose impairments precipitated by antipsychotics.

#### 4.3.6 Antidepressants

With an increased prevalence of depression and associated disorders, an increase in the prescription of antidepressants is inevitable. Various *in vivo* and *in vitro* studies indicated that antidepressant therapy led to increased oxidative stress and mitochondrial dysfunction (6, 83–86). Amitriptyline is one of the widely prescribed tricyclic antidepressants and has been demonstrated to induce mitochondrial toxicity through numerous mechanisms. These include reduced mitochondrial content, mitochondrial protein expression an increase in mitochondrial membrane permeability transition, and the generation of ROS (67). Other antidepressants such as citalopram, amitriptyline, and imipramine were observed to inhibit complex I in isolated pig brain mitochondria. A study by Abdel-Razaq et al. (83) demonstrated that 18-hour exposure to clomipramine, desipramine, and norfluoxetine promotes apoptosis in the CHO Beta SPAP cell line and pig heart *in vitro*. These observations were associated with a decrease in mitochondrial integrity as well as Complex I, II, III and IV activities (83). On the contrary, other studies have reported an increase in mitochondrial function *in vivo* after a week of antidepressant administration (85). However, there has been increasing evidence of a decrease in mitochondrial function with antidepressant exposure. Clomipramine and impramine have been shown to possess anti-cancer effect which is attributed to their ability to increase apoptosis in tumour cells (84). Conflicting data has been reported regarding the effect of antidepressants on insulin sensitivity. A study conducted by Levkovitz et al. (86) intended to explore mechanisms involved in clinical metabolic syndrome induced by antidepressants, particularly selective serotonin re-uptake inhibitors (SSRI) (86). In this study, paroxetine and sertraline attenuated the insulin activation of IRS-1 which was associated with phosphorylation and activation of Ser/Thr IRS-1 kinase except PKB. The observations implicated antidepressants in instigating insulin resistance. However, it was not clear whether it was a direct effect on the insulin signalling pathway or mitochondrial dysfunction Therefore, this calls for more studies aiming at exploring a link between antidepressant-induced-mitochondrial toxicity and risk of developing insulin resistance.

#### 4.3.7 Antibiotics

Antibiotic therapy undeniably represents an invaluable asset to fight bacterial infections as it is the first line of therapy. Antibiotics have significantly contributed to the reduction of bacterial infection-related morbidity and mortality rates (26, 87–89). However, prolonged use of antibiotics has been linked to severe adverse events such as disruption of the microbiome, nephrotoxicity, chondrotoxicity, tendinopathy, and mitochondrial impairment being central (26, 85). Mitochondrial evolved from endosymbiotic alpha-proteobacteria, hence making it similar to bacteria in numerous ways (85, 86). The mitochondria and their components may therefore be an antibiotic target site due to their endosymbiotic nature. Various classes of antibiotics such as macrolides, aminoglycosides, and amphenicols inhibit bacterial protein synthesis, they also block polypeptide synthesis in the mitochondria (85, 86). A variety of aminoglycoside’s adverse effects such as ototoxicity, kidney injury and vestibular toxicity are hallmarks of mitochondrial toxicity (87). A macrolide such as clarithromycin and azithromycin exert their pharmacological effect by binding within the exit tunnel of the bacterial ribosome and perturbing ribosomal translocation. Consequently, clarithromycin also inhibits mitochondrial protein synthesis (88, 89). Previous research studies reported that other classes of antibiotics such as quinolones (ciprofloxacin), aminoglycosides (kanamycin), and ß-lactams (ampicillin) induce oxidative stress leading to the overproduction of ROS that perturbs mitochondrial function (90). The clinical effect of these antibiotics was dose and time-dependent. Increased ROS production can damage mitochondrial DNA and genomics, especially in individuals with mutated antioxidant genes resulting in reduced mitochondrial content (87). Various studies support the hypothesis which states that a decrease in skeletal muscle mitochondrial content contributes to the development of insulin resistance (89). In an *in vivo* study performed by Frederico et al. (91) an increase in mitochondrial content in human skeletal muscle (measured by citrate synthase activity and cardiolipin content) reported improved hyperglycaemia in T2D patients. However, Phielix et al. (92)found no correlation between insulin sensitivity and mitochondrial content. Doxycycline is a tetracycline antibiotic that has been reported to inhibit mitochondrial protein synthesis and lead to a decreased mitochondrial energy-generating capacity (43, 44). Additionally, Dijk et al. (43) reported that doxycycline exposure causes oxidative stress and lowers mitochondrial membrane potential. Chen et al. (44) performed an *in vivo* study using mice models and 20 μg/mL of doxycycline improved glucose tolerance significantly and decreased fasting blood glucose. The percentage of larger islets (diameter>350µm) and islets mass increased in the presence of 200 μg/mL doxycycline. The study reported a significant decrease in lipopolysaccharide levels and increased beta-cell ratio in all mice models treated with doxycycline. Chen et al. (44) concluded that doxycycline improves islets morphology and glycaemic control even at extremely low concentrations. On the contrary, compelling evidence suggests that mitochondrial impairment or a decrease in mitochondria number after antibiotic exposure predisposes individuals to obesity. However, the direct link between antibiotics and weight gain remains elusive (43). One of the most common features of obesity is insulin resistance which leads to the accumulation of excessive lipids and their secondary metabolites in metabolically active tissues. Therefore, it is plausible to suggest that prolonged antibiotic exposure can lead to impaired metabolic fuel oxidation, decreased mitochondrial oxidative capacity, and accumulation of lipotoxic lipid intermediates culminating in the development of insulin resistance. A population-based case-control study conducted in Denmark concluded that the odds ratio associating antibiotic exposure to T2DM was 1.53 (95%confidence interval 1.50–1.55) (93).

#### 4.3.8 Antihypertensives

Several classes of antihypertensive drugs such as angiotensin-converting enzyme inhibitors (ACE inhibitors), calcium channel blockers, and beta blockers are used in the management of hypertension. Additionally, combination therapy of antihypertensive drugs was found to be more efficient especially in severely hypertensive patients however, adverse effects have been reported (48, 94–96). A study conducted by Ziegelhöffer et al. (48) demonstrated that nifedipine and captopril altered mitochondrial function in hypertensive rats. Similarly, a study conducted by Kancirová et al. (94) demonstrated that captopril decreases the activity of the ATP synthase enzyme in the mitochondria and slightly increases fluidity in the mitochondrial membrane. Interestingly the inhibitory effect of captopril was reduced when combination therapy was administered (94). Various studies reported that insulin-stimulated glucose utilisation significantly decreases in the peripheral tissues of hypertensive patients. However, majority of ACE inhibitors improve insulin sensitivity (48, 94). An *in-vivo* study conducted by Loloi et al. (95) demonstrated improved insulin sensitivity following chronic captopril exposure. Previous *in vitro* studies conducted reported that lipophilic ß blockers such as metoprolol inhibited the 3^rd^ stage of mitochondrial cellular respiration (96, 97). The direct effect exerted by ß blockers on the mitochondria could explain the chronic fatigue experienced by some patients on treatment (96, 97). Sakurada et al. (96) reported that oxidative phosphorylation and oxidation of substrates combined with NAD+ were inhibited in the presence of propranolol. There was also a reduction in the mitochondrial transporters and enzyme activity (96). Carvedilol is a non-selective ß blocker with α-1 blocking activity. Carvedilol has antioxidant properties and inhibits complex 1 respiratory chain, ATPase, and stage 3 respiration (45–47, 98). Carvedilol also induces a mild uncoupling effect in mitochondrial oxidative phosphorylation (45, 98). In addition to its cardiac effect, Ferrua et al. (45) conducted a study with non-diabetic cardiac heart failure patients and noted an improvement in insulin sensitivity (45).

#### 4.3.9 Anti-epileptics

The anti-epileptic drugs (AEDs) exhibit beneficial and adverse effects on the function of the mitochondria. Valproic acid (VPA) is a commonly used anti-epileptic drug that has a well-established side effect of hepatotoxicity and insulin resistance (50, 99–101). Currently, there is no established mechanism for how hepatotoxicity develops. However previous research studies observed a strong inhibition of pyruvate oxidation in liver mitochondria in the presence of valproic acid (50, 51, 99–101). Valproic acid depletes carnitine in the liver resulting in decreased ß-oxidation (45). ß-oxidation plays a significant role in lipid metabolism such that its inhibition results in the accumulation of lipids in hepatocytes (50, 99). Previous animal studies performed reported an increase in mitochondrial protein and peroxisome proliferation. However, impaired mitochondrial ß-oxidation of fatty acids was observed which could have a negative effect on insulin sensitivity (99). Interestingly, in an *in vivo* study of 3-nitropropionic acid-induced seizure in mice, VPA reversed mitochondrial toxicity in the respiratory chain (51). Phenytoin is a sodium channel blocker that is commonly prescribed as an anti-seizure drug. However, phenytoin has been reported to decrease intracellular glutathione, increase ROS production, and amplify lipid peroxidation and mitochondrial impairment in rat hepatocytes (51, 69, 102). Interestingly, an injection of phenytoin intraperitoneally increased the activity of superoxide dismutase and decreased a biomarker of oxidative stress known as cerebral malondialdehyde (51). Carbamazepine (CBZ) is a dibenzoazepine with structural similarities to tricyclic antidepressants. CBZ is the first-line therapy for partial or generalized tonic and mixed convulsions (52, 103). CBZ has been associated with mitochondrial toxicity and an *in vivo* study conducted by Alelwani et al. (52) demonstrated that at low concentrations of 0,1 to 10µM CBZ significantly inhibited mitochondrial complex I and III upon 3 hours of exposure.0,1µM significantly inhibited both complexes after 24 hours of exposure. Im et al. (53) reported that CBZ promoted differentiation in adipocytes and increased levels of insulin signalling proteins such as insulin receptor (IR), insulin receptor substrate (IRS), GLUT 4, and Akt. Interestingly, CBZ exposure in differentiated adipocytes did not increase the expression of Akt phosphorylation (53).

#### 4.3.10 Analgesics

Acetaminophen (paracetamol, N‐acetyl‐p‐aminophenol; APAP) is a commonly used drug due to its analgesic and antipyretic effects (55, 56, 104). Previous research studies reported that high doses of APAP inhibited complex I and II in mouse hepatocytes however, there is inconclusive evidence in human studies (56, 57). Paracetamol is absorbed from the gastrointestinal tract and is metabolized by cytochrome P‐450 (CYP) enzymes in the kidney and liver and forms a reactive metabolite known as N‐acetyl‐p‐benzoquinone imine (NAPQI) (56). NAPQI is effectively detoxified by hepatocellular glutathione. However, high doses of APAP deplete glutathione levels resulting in the formation of protein adducts and eliciting oxidative stress in the mitochondria (56, 57). The protective system is effective when low levels of protein adducts are formed and therapeutic doses are administered (56). Various animal studies demonstrated that low doses of paracetamol potentially lower blood glucose levels in several animal models including diabetic and high-fat (HF) diet-induced models (57, 58). Extensive laboratory and pre-clinical studies have reported that paracetamol off-label applications may be derived from its ability to function as an antioxidant (57, 58). Shertzer et al. (58) reported that paracetamol at 20 or 30 mg/kg body weight prevents hyperglycaemia in high-fat diet animal models. Additionally, paracetamol also improved glucose tolerance, increased the ability of the HF-fed mice to boost blood insulin levels, and restored fasting insulin levels (58). Although the exact mechanism(s) involved remain unclear, these effects have been postulated to arise due to improved pancreatic insulin synthesis/secretion. However contrary evidence suggested that lethal concentrations of paracetamol-induced hyperglycaemia rapidly. Clark et al. (103) demonstrated that paracetamol overdose can potentially impair peripheral glucose uptake. According to Mohamed et al. (105) physiological pH tramadol presents a positive charge such that it can potentially accumulate within cell compartments that are negatively charged such as the mitochondria. Therefore, tramadol diminishes the activities in mitochondrial complexes I, III, and IV (59, 105, 106). The study was conducted using a dose range between 42 to 168 mg/kg for 30 days, this led to the formation of ROS in the mitochondria and induced oxidative stress (105). The protein/lipid ratio in the mitochondrial inner membrane is 3:1 thus upon tramadol exposure lipoxidative and protein oxidation damage can be induced by ROS (59, 60, 105, 106). Choi et al. (59) demonstrated that tramadol increased glucose utilization *via* direct activation of MTOR in isolated hepatocytes and skeletal muscles of streptozotocin-induced diabetic rats. Ghosh et al. (61) reported that 5µM and 10 µM of diclofenac did not affect complex I activity however 5µM significantly impaired complex III in isolated heart mitochondria. Inhibition of complex I, II, and III have been associated with the production of mitochondrial ROS (107). In a study conducted by Sun et al. (107) a significant increase in serum insulin and reduced HOMA-IR was reported, indicating that it can improve insulin resistance. Diclofenac was also reported to improve dyslipidaemia and hyperglycaemia associated with diabetes mellitus (107).

#### 4.3.11 Anticoagulants

Antiplatelet drugs exhibit their adverse effects *via* numerous mechanisms that are not fully known yet. However, the mitochondria are one of the target sites for toxicity (63, 64). Acetyl-salicylic acid (ASA) is a drug that is commonly prescribed worldwide as an antithrombotic agent, and it also has analgesic and anti-inflammatory properties (63, 64, 108). ASA has been on the market for decades, however, in previous *in-vitro* studies, it exhibited significant mitochondrial toxic effects. ASA reduced ATP output in rat liver by inhibiting respiratory chain ATPase in rat liver mitochondria (64). An *in-vitro* study conducted by Al-Nasser et al. (109) demonstrated that ASA potentiates the opening of mitochondrial transition pores resulting in a decreased mitochondrial membrane potential and release of calcium from the mitochondria which eventually leads to apoptosis. Sun et al. (107) reported improved insulin sensitivity in aspirin-treated rats with suggestions that aspirin improves insulin sensitivity by inhibiting hepatic NF-κB activation and TNF-α level.

Clopidogrel is a P2Y_12_ inhibitor that is widely prescribed in antiplatelet therapy mainly in the management of cardiovascular diseases (110–112). Tai et al. (111) conducted an *in-vitro* study and observed a reduction in mitochondrial respiratory state 3 and state 4 respiration and prolonged oxygen consumption in State 3 in the presence of high doses of clopidogrel. This observation indicated a disruption in mitochondrial oxidative phosphorylation. However, therapeutic doses administered in humans did not exhibit any mitochondrial respiratory chain impairment (113). Clopidogrel reactive metabolites depleted cellular glutathione content in HepG2 cells and primary human hepatocytes leading to the accumulation of ROS and mitochondrial impairment (65, 113). A study conducted by Maseneni et al. et al. (113) in human lymphocytes and neutrophil granulocytes observed a reduction in inner mitochondrial membrane potential, induced release of cytochrome c, and enhanced production of ROS in the presence of clopidogrel. Interestingly a study conducted by Mohamed et al. (65) demonstrated an improvement in insulin sensitivity and a reduction in serum insulin levels in diabetic patients consecutively taking clopidogrel and oral hypoglycaemic agent after 2 months of therapy. The mechanism through which clopidogrel lowers glucose levels is not yet established however, it can be associated with the inhibitory effect of clopidogrel on TNF-a production.

## 5 Authors’ perspective and recommendations

As mentioned above, the prevalence of diabetes mellitus is still on the rise, despite preventative strategies in place, and this will place a huge burden on the healthcare systems. Therefore, it is imperative that we accelerate our understanding of the risk factors associated with the development of the disease. Over the years, we have established that the elevation of free fatty acids have a negative effect on insulin sensitivity. Recently, we have seen an acceleration of research with much focus on mitochondrial dysfunction as a risk factor for developing insulin resistance. Although few studies have demonstrated a weakened insulin sensitivity upon mitochondrial toxicity, it has not been clear how these are mutually linked. Mitochondrial toxicity has been well recognized to affect the utilisation of substrates with the accumulation of toxic lipid metabolites such as diacylglycerol and ceramide. These lipid metabolites have been shown to negatively affect PI3-K and Akt activity, thus compromising the insulin signalling pathway. As previously alluded, many pharmacological agents induced mitochondrial toxicity and insulin resistance development have been reported. Despite a positive association between drug-induced mitochondrial toxicity and glucose impairments observed with some pharmacological agents, however, it is not true for some agents as an improvement in glucose is seen despite mitochondrial dysfunction i.e some antihypertensives and analgesics presented above. This therefore could mean our understanding of mitochondrial toxicity and insulin resistance is limited

A further conflict is supported by the fact that some antidiabetic drugs such as biguanides and thiazolidinediones are known inhibitors of mitochondrial complex I and II, however, these are conventional insulin sensitizers used in clinical settings to date. This observation raises some questions, i.e., does suppressing specific functions of the mitochondria associate with improvement in insulin sensitivity? Also, other questions that should be addressed are the extent and duration of the mitochondrial toxicity associated with insulin resistance. Also, although different pharmacological agents can instigate similar mitochondrial toxicities, they could present with other biological properties that can perhaps mask the negative impact of mitochondrial toxicity on insulin signalling regulation. Therefore, we cannot blindly associate any drug with risk of developing insulin resistance by the virtue of its negative impact on mitochondrial function. Furthermore, conflicting observations could also be attributed to different experimental approaches, therefore there is a need to probe experimental paradigms utilised by different researchers. These observations in part further allude to and acknowledge the complexity of mitochondrial function and its influence on biochemical pathways such as glucose handling by insulin-sensitive cells. This, therefore, suggests more studies are needed to demonstrate a clear relationship between mitochondrial function/dysfunction and insulin sensitivity beyond a reasonable doubt. Perhaps we can achieve this by striving to develop insulin models using mitochondrial toxicological agents’ exposure. In this way, we can characterise the models to determine the biochemical pathways activated and suppressed leading to insulin resistance. Additionally, the reversal of insulin resistance through attenuating mitochondrial toxicity should be observed in such models. This would allow us to further expand our understanding on the pathogenesis of insulin resistance and involvement of the mitochondrial toxicity.

Despite conflicting observations, we have presented in this review, we believe we have highlighted substantial evidence to consider drug-induced mitochondrial toxicity as a risk factor for developing insulin resistance. Hence more direct studies are required to elucidate the mechanisms in insulin-sensitive cells such as the liver, adipose tissue, and skeletal muscle. The observation from these studies and correlation could perhaps provide other neglected risk factors that predispose individuals to insulin resistance, ultimately diabetes. Our understanding could therefore assist in developing strategies to mitigate these risks. Unfortunately, some of these pharmacological modalities are life-saving and chronic medications, therefore, they cannot be discontinued until safer medicines are at our disposal. Although research and development strive to find safer medicines, the reality is that mitochondrial toxicity remains for most. Therefore, it’s imperative that we devise strategies with the intention to attenuate the risk of developing insulin resistance through drug-induced mitochondrial toxicity. Firstly, we could educate patients taking these therapies to be more physically active and adopt healthy diets. Secondly, we could also possibly investigate therapies that would be administered as adjuvants with these agents to attenuate mitochondrial toxicity. Many supplements have been reported to be beneficial against mitochondrial toxicity, hence clinicians should consider and assess its benefits. Thirdly, communities need to be educated about such risks, as some of the medications are easily accessible, yet associated with such risks. Also, this could perhaps suggest that even tighter regulations are required for medicines associated with mitochondrial toxicity, especially for over-the-counter medications. Fourth, research and development towards screening for mitochondrial toxicity should be accelerated. Thus, there is a need to find sensitive and effective technology to screen for mitochondrial toxicity for frequent testing. This would allow accelerated detection and monitoring of mitochondrial dysfunction. Lastly, the issue of polypharmacy can also contribute immensely to this drug-induced mitochondrial toxicity. Most geriatric patients are on polypharmacy, and they are also predisposed to reduced mitochondrial function due to their age. Polypharmacy can therefore increase the risk of being exposed to multiple mito-toxicological at once thus increasing the risk of developing insulin resistance further. Therefore, it is imperative to understand the implications of polypharmacy on mitochondrial impairments and the risks of developing insulin sensitivity impairments.

## 6 Conclusions

Substantial evidence supports that a variety of drug classes and environmental toxicants can induce mitochondrial dysfunction through increased ROS production, mtDNA depletion, and uncoupled OXPHOS. Since the discovery of mitochondrial dysfunction in the 1960s, the central role mitochondria play in health and disease has been widely documented. Mitochondrial impairment is now understood to play a significant role in a wide range of seemingly unrelated disorders such as DM, schizophrenia, chronic fatigue syndrome, Parkinson’s disease, and non-alcoholic steatohepatitis. In this review, we have demonstrated that numerous drugs associated with mitochondrial toxicity instigate glucose handling impairments. However. whether these mitochondrial defects represent a cause or a consequence of insulin resistance in skeletal muscle remains to be fully elucidated.

## Author contributions

Conceptualization: AKu, NS, AKh, PN, MM, CA. Original draft preparation: AKu, NS. Writing-review and editing: AKu, CA, NS, AKh, PN, MA. Supervision: NS, MM, PN, AKh. All authors contributed to the article and approved the submitted version.
